# Implementation of the Recovery Guide in inpatient mental health services in Sweden—A process evaluation study

**DOI:** 10.1111/hex.13480

**Published:** 2022-03-27

**Authors:** Ulrika Bejerholm, Conny Allaskog, Jessica Andersson, Linda Nordström, David Roe

**Affiliations:** ^1^ Department of Health Sciences, Centre of Evidence‐Based Psychosocial Interventions, CEPI Lund University Lund Sweden; ^2^ Department of Research, Development and Education Division of Psychiatry and Habilitation Lund Sweden; ^3^ The Swedish Partnership for Mental Health, NSPH Skåne Sweden; ^4^ Department of Community Mental Health University of Haifa Haifa Israel; ^5^ Department of Clinical Medicine, Psychiatry Aalborg University Aalborg Denmark

**Keywords:** coproduction, mental health, person‐centred, process evaluation

## Abstract

**Background:**

Involving service users in inpatient care and recovery planning has gained interest worldwide. Our purpose was to evaluate the process of implementation of a coproduced Recovery Guide (RG) intervention in 22 inpatient wards in Sweden, in terms of context, implementation process and mechanisms of impact over 12 months.

**Methods:**

A mixed method design and a process evaluation framework were used to guide data collection and to deductively analyze perspectives and descriptive statistics of delivery from three stakeholder groups.

**Results:**

Results showed that although initial contextual barriers were present (e.g., lack of resources, and interest, uncertainty in the organization, a dominant illness perspective), it was possible to implement the RG in 14 wards, where 53% of admitted service users received the intervention. Legitimacy of the intervention, engaged managers and staff, capacity of staff and ward organization, coproduction and continuous support from user organization were critical mediators. Mechanisms of impact concerned (1) a new perspective on mental health, well‐being and recovery, (2) capacity building of a recovery approach in inpatient settings and (3) a meaningful outlet for users' thoughts and feelings on recovery, sharing narratives and influencing care and goals.

**Conclusions:**

The RG intervention has the potential to promote a recovery approach in inpatient mental health services (MHSs). Coproduction among stakeholders created trust and a sustainable implementation that made it possible for wards to resume implementation when contextual barriers had been resolved.

**Patient and Public Contribution:**

The current study involved stakeholders including a service user organization, the public, first‐line managers and staff (including peer support workers) in inpatient and community MHS and researchers, who greatly contributed to the implementation programme, including codesign of the RG intervention as well as coproduction of the implementation in inpatient MHS. All authors have their own lived experiences of mental health problems as a service user or as a relative.

## 1 INTRODUCTION

The concept and practice of personal recovery are critical to advance when moving towards more positive psychiatry, that is, mental health services (MHSs) encompassing service users' optimism, resilience, coping, self‐efficacy, social engagement and meaning in life even when living with the consequences of having mental illness.^1^ While the importance of addressing clinical treatment together with personal recovery has been recognized in modern MHSs, the implementation of person‐centred treatment planning remains challenging.^2^, ^3^ Although Swedish national policy advocates person‐centredness and thus recovery‐oriented services,^4^ they are seldom implemented.^5^, ^6^ Critical barriers have been identified on different levels of the organization, as well as in relation to access to continuous implementation support.^7^ Recovery‐oriented interventions may contradict traditional care paradigms in values and perspectives, at both the organizational (meso) and front‐line operational (micro) levels.^1^ Attempts have been hindered by uncertainty at these levels of how to implement such new perspectives and interventions into regular work cultures and routines.^8^

Inpatient MHSs focus on treatment and acute crises and challenges. Yet it might be precisely during such times that recovery‐oriented services are the most crucial when users need the most support in trying to make sense, process, cope and remain hopeful.^9^, ^10^ In addition, it might be a time crucial to empower and assure users' involvement in care decisions,^2^ and control and involvement in upcoming events in everyday lives.^11^ Nevertheless, inpatient wards can be particularly challenging settings to promote a recovery‐focus.^2^, ^12^, ^13^ In a Norwegian survey, including managers and staff from 83 inpatient wards, it was reported that MHSs lacked a reflective culture and knowledge of how to empower service users.^14^ Staff had limited resources, a high workload and felt pressure, which fostered stereotypical views on care and service users. In Sweden, inpatient staff perceived that they had limited time for reflection and sharing experiences with colleagues, and thus expressed a desperate need for resources and strategies to meaningfully connect with users.^15^ Likewise, staff in Australia emphasized the need for a clear description and support on how to become recovery‐oriented.^12^ A review of recent literature clearly emphasizes that users and staff need tools to enhance common ground and that recovery values and interventions are needed in inpatient MHS.^2^ In response to this knowledge and practice gap, we developed the Recovery Guide (RG) intervention to support the implementation of a recovery‐oriented approach in inpatient MHS.

The growing awareness of the importance of meaningfully involving service users and user organizations in the augmentation of MHS has evolved in response to critiques of traditional care.^1^, ^16^, ^17^, ^18^ User influence is further decisive to develop and evaluate sustainable interventions that are meaningful and helpful.^18^, ^19^ Coproduction has become a hallmark of a recovery‐oriented MHS.^20^ Researchers may follow the same participatory trend and contribute to shaping and performing research planning and implementation in a more collaborative and less hierarchical manner. Accordingly, the MHSs of Region Skåne, the user organization of NSPH Skåne (The Swedish Partnership for Mental Health) and the research network of Centre of Evidence‐based Psychosocial Interventions (CEPI)^21^, ^22^ have collaborated to advance the process evaluation of the RG. Furthermore, to design and evaluate complex interventions, whether it is feasible to implement in a certain context, process evaluation is an essential part.^23^

The purpose of the present study was to investigate the implementation of the RG intervention in inpatient MHS settings that was part of their regular quality improvement. More specifically, guided by the Medical Research Council (MRC) process evaluation framework,^23^ our aim was to gain a better understanding of the process evaluation in terms of inpatient MHS context, implementation process and mechanisms of impact.

## 2 MATERIALS AND METHODS

### 2.1 Research design and inpatient settings

According to the MRC framework,^23^ the intervention and the implementation programme were evaluated in reflection of the implementation process, as it occurred. The method and result descriptions are in accordance with reporting process evaluations. The study took place during the year of 2019 and 22 inpatient wards from all four subunits of the MHSs in the Region Skåne in Sweden partook. Six were acute inpatient wards, others specialized in diagnoses, that is, psychosis, affective and other mental health disorders, alcohol and substance abuse or eating disorders. All first‐line managers (*n* = 22) agreed to participate. Staff participating in focus group interviews (*n* = 32) were recruited by means of convenience sampling as they worked in shifts. For the brief survey about the RG intervention (after Education Step 1), 139 staff participated. Furthermore, 33 users anonymously filled in a survey. In all, 226 participants partook in the data collection.

Informed consent was obtained by all participants. The NSPH informed staff and managers about the purpose, and terms and use of data collection, for example, that it complied to general data protection regulation, was voluntary, would be analyzed to facilitate on‐going implementation and reported to stakeholders in relation to the quality improvement in MHS. Staff informed inpatient users about the same, who were invited as experts acting in an advisory capacity. They were asked to complete a paper–pencil survey about their perception of the RG intervention and put it in a closed envelope. Staff gathered all material and handed it to NSPH. All participants were informed that they consented to participation as they entered data collection, that data was anonymized and securely stored and that they could decline participation anytime. Researchers had no contact with participants and were subjected to secondary data that were available to the public and revealed no sensitive or personal information. Pulling together data sets during analysis neither posed  risk of deanonymizing participants. Furthermore, the intervention was the subject for data collection and not the human, that is, the questions posed concerned the use of the intervention and posed limited risk, harm or inconvenience for participants. Since nonpersonal and nonsensitive data was aggregated in these regular quality development activities of MHS, minimal harm was done.

### 2.2 The RG intervention

The RG intervention regards the introduction and follow‐ups of *The Recovery Guide—For You in Inpatient care*.^24^ The RG material is available as a booklet, which includes an introduction and nine chapters: (1) To be admitted, (2) Your recovery, (3) Everyday life during inpatient stay, (4) Your rights as service user, (5) Safety plan, (6) Reflections on challenges, (7) Well‐being factors, (8) Caring for your physical well‐being and (9) Before discharge. Brief narratives of lived experience constitute the core pedagogic material to help users relate to their vision of recovery. The narratives are diverse, yet give voice to some growth processes, to provide reflections and hope. A manual for staff explains each chapter, views on recovery, suggestions on introduction, typical questions and answers, as well as delivery guidelines on an early introduction (first 1–5 days), registration on usage and treatment and care planning in medical records.

The springboard for the development was the Danish self‐help project named ‘Discharged’ in which a digital material ‘When you are discharged’ is designed to minimize frictions between inpatient and outpatient MHSs and prepare for discharge.^25^ The development was further informed by the workbook ‘Mapping our madness’, developed through the peer‐based social media network called the Icarus project in New York,^26^ the Crisis Plan in the Wellness Recovery Action Plan^27^ and the NHS Taking Back Control.^28^

The development was coproduced among stakeholders representing reference groups, unit‐ and first‐line managers and staff (Table 1) from all four subunits (A–D, Table 2). Emerging structures were built on inclusive and transparent decision processes at different organizational levels.^29^ While the NSPH orchestrated the development, reference groups and one referral group that represented the public critically informed the iterative design process.^24^ CEPI coproduced implementation programme and design of process evaluation. In all, 120 persons partook.

**Table 1:** Implementation programme of the Recovery Guide intervention

Components of implementation programme	Content	Time
Preparatory phase	‐ Decision by the head of MHS and NSPH user organization to coproduce the RG and to implement it in inpatient care	12 months before
Introductory phase	‐ Introduction to the concept of recovery and RG to subunit managers, first‐line managers and staff ‐ Cocreation with reference groups with stakeholders from all inpatient subunits A–D ‐ Coproduction with reference groups on delivery and feedback guidelines regarding the distribution of responsibilities, checklists and progress report documentation ‐ Identifying RG responsible persons	6–12 months before
Reflection phase	‐ Participatory meetings regarding the RG material and implementation with reference groups, referral groups including users and relatives in inpatients care, peer‐support workers, staff and users from community MHS and researchers	3–6 months before
Pre‐education phase	‐ NSPH educating the educators with previous experience of RG delivery ‐ Providing education templets, checklists and progress report sheets to educators to use in their teaching	
Education, Step 1	‐ Education at 8 inpatient wards and the start of delivery	0–2 months
Reflection phase	‐ Participatory meetings on feedback from education and RG intervention delivery and final adjustments	3 months
Education, Step 2	‐ Education at 14 inpatient wards and the start of delivery	2–4 months
Continuous supportive feedback	‐ Monthly site visits to all 22 wards. Dialogue about RG delivery, supportive feedback about responsibilities, checklists and progress report documentation	2–12 months
Reporting key‐findings	‐ Summarizing and reporting key findings to stakeholders for quality improvement	12 months

Abbreviations: MHS, Mental Health Service; RG, Recovery Guide.

**Table 2:** Delivery of the Recovery Guide intervention during the implementation period

Inpatient unit	Subunit	Ward type	May	June	July	August	September	October	November	December		
			Delivered (*n*)	Delivered (*n*)	Delivered (*n*)	Delivered (*n*)	Delivered (*n*)	Delivered (*n*)	Delivered (*n*)	Delivered (*n*)	Mean % of dose delivered/received, (min–max), range (*R*)Yes/No	Ongoing delivery during study period
			Admitted (*n*)	Admitted (*n*)	Admitted (*n*)	Admitted (*n*)	Admitted (*n*)	Admitted (*n*)	Admitted (*n*)	Admitted (*n*)		
			Percentage delivered of admitted (%)	Percentage delivered of admitted (%)	Percentage delivered of admitted (%)	Percentage delivered of admitted (%)	Percentage delivered of admitted (%)	Percentage delivered of admitted (%)	Percentage delivered of admitted (%)	Percentage delivered of admitted (%)		
1	A^a^	Psychosis	20, 42, 48%	15, 56, 27%	13, 33, 39%	12, 37, 32%	27, 39, 69%	13, 37, 35%	9, 37, 24%	12, 33, 36%	*m* = 39% (24–69), *R* = 45	Yes
2	A^a^	General	4, 26, 15%	8, 22, 34%	15, 31, 48%	7, 39, 18%	4, 42, 10%	8, 32, 25%	8, 29, 28%	8, 22, 34%	*m* = 26% (10–48), *R* = 38	Yes
3	A	General	3, 16, 19%	7, 21, 30%	0, 27, 0%	2, 38, 5%	–	5, 29, 17%	1, 19, 5%	1, 26, 4%	*m* = 11% (0–30), *R* = 30	Yes
4	B^b^	Psychosis	–	–	–	–	–	6, 7, 86%	–	–	–	Yes
5	**B** a	**General**	**19, 37, 51%**	**14, 20, 72%**	**17, 31, 55%**	**13, 15, 87%**	**13, 16, 81%**	**18, 20, 90%**	**15, 25, 60%**	**11, 14, 79%**	***m*** = **72% (51**–**90), *R*** = **39**	**Yes**
6	B^c^	General	–	–	–	–	–	–	–	‐‐	–	No
7	B	General	7, 40, 18%	16, 26, 62%	5, 30, 7%	5, 35, 14%	20, 32, 62%	6, 32, 19%	10, 30, 33.57%	18, 34, 53%	*m* = 34% (7–62), *R* = 55	Yes
8	B^b^	Psychosis	–	–	–	–	–	–	0, 32, 0%	2, 29, 7%	–	Yes
9	B^a^^,^ b	Psychosis	–	–	–	–	–	–	–	–	–	No
10	B	General	18, 31, 58%	21,44,48%	9, 32, 28%	15, 23, 65%	10, 23, 43%	20, 44, 45%	20, 45, 44%	15, 27, 56%	*m* = 48% (28–65), *R* = 37	Yes
11	**B**	**Substance use**	**14, 28, 50%**	**14, 28, 50%**	**14, 28, 50%**	**14, 28, 50%**	**20, 25, 80%**	**18, 24, 75%**	**27, 28, 98%**	**16, 21, 76%**	***m*** = **66% (50**–**98), *R*** = **48**	**Yes**
12	B	Substance use	15, 51, 29%	18, 56, 32%	12, 65, 18%	10, 58, 17%	9, 46, 20%	15, 60, 25%	22, 60, 37%	9, 37, 24%	*m* = 25% (17–37), *R* = 20	Yes
13	B^d^	Intensive care	6, 18, 33%	–	–	–	–	–	–	–	–	No
14	**C**	**General**	**35, 41, 85%**	**36, 41, 88%**	**36, 41, 88%**	**38, 43, 88%**	**35, 38, 92%**	**32, 39, 82%**	**35, 45, 78%**	**11, 11, 100%**	***m*** = **88% (78**–**100), *R*** = **22**	**Yes**
15	C^b^	Elderly	5, 27, 19%	5, 20, 25%	10, 18, 56%	8, 17, 47%	9, 16, 56%	8, 17, 47%	12, 15, 80%	8, 17, 47%	*m* = 47% (19‒80), *R* = 61	Yes
16	C^d^	Substance use	–	–	–	–	–	–	–	–	–	
17	**C** d	**General**	**45, 46, 98%**	**45, 46, 98%**	**45, 46, 98%**	**47, 47,100%**	**44, 46, 92%**	**45, 46, 98%**	**41, 41, 100%**	**40, 40, 100%**	***m*** = **98% (92–100), *R*** = **8**	**Yes**
18	**C** a ^,^ b	**Psychosis**	**27, 40, 68%**	**32, 40, 80%**	**28, 42, 67%**	**31, 45, 69%**	**18, 28, 64%**	**25, 42, 60%**	**27, 40, 68%**	**30, 46, 65%**	***m*** = **76% (60–80), *R*** = **20**	**Yes**
19	D^e^	Not specified	1, 12, 8%	–	–	–	–	Closed	–	–	–	No
20	D^d^^,^ e	Eating disorder	–	–	–	–	–	–	–	–	–	No
21	D^d^^,^ e	Not specified	–	10, 53, 19%	22, 47, 47%	6, 33, 18%	Closed	–	–	–	–	No
22	D^a^^,^ c ^,^ e	Not specified	–	–	–	–	–	–	–	–	–	No

*Note*: Rows in bold indicate wards with a mean delivery rate >50%.

^a^ Staff educated in Step 1 (Table 1).

^b^ Dates were set for continuing education sessions in 2020.

^c^ Transformed into daycare centre.

^d^ New dates were set for education and resumption of implementation in 2020.

^e^ Wards subjected to reorganization.

### 2.3 The implementation programme

Implementation planning in different steps forms a critical part of quality implementation.^30^ The implementation programme is described in Table 1. The Preparatory phase aimed to facilitate the adoption of the intervention, a buy‐in from stakeholders to foster a supportive implementation climate. The Introductory phase concerned reaching consensus and building the general capacity of a proactive implementation support system and structure for delivery, while the Reflection phase regarded public involvement and alteration to enhance acceptability and user‐friendliness. Pre‐education was concerned with recruitment and training of educators, for example, pedagogic form, materials and format of delivery. The Education steps and the Reflection phase focused on final adjustments of education and material. Final phases of continuous supportive feedback and reporting key‐findings helped to share barriers and facilitators that unfolded over time, verbally during monthly visits and as a written report.

The programme entailed implementation in 22 wards. Firstly in eight wards, and secondly in the remaining 14 wards. Including stakeholders at both meso‐ and microlevels of the organization helped to mitigate uncertainties that can arise in both vertical and horizontal relationships.^31^ Coproduction and social networking aimed to enhance both informal and formal communication during all phases of implementation.^32^ Education was a critical component to assure that staff had competencies relevant for delivery.^30^ Staff from the same ward were educated together. No specific discipline was sought, but staff whom users met on a daily basis were represented, for example, peer supporters, nurses, assistant nurses, social workers and medical doctors. The education session was administered to 3 h and concerned content, discussions about preconceptions, a role play to practice mutual dialogue (listening, addressing needs), and how to personalize delivery. A home assignment preceded the training.

### 2.4 The process evaluation plan

A convergent parallel mixed methods design was used to study the process evaluation components, using both qualitative and quantitative data.^33^ It helps to understand complex research problems and questions posed in MHS research.^34^ The *context* component refers to factors that affect intervention, implementation and potential outcomes. The *implementation process* focuses on the dose delivered or received, the target group reached and possible adaptations. *Mechanisms of impact* focus on how participants respond to interactions with interventions.^23^

Data from managers and staff were collected by C. A., J. A. and L. N. from NSPH (with support from CEPI), who all have previous experience of this in MHS settings. Initial data collection covered the implementation of eight wards and occurred in connection to Education Step 1 (Table 1), where 139 staff completed a brief pen–paper survey about the RG intervention and education. At 3 months follow‐up, during the Reflection phase, six focus group interviews with staff (*n* = 32) and individual interviews with first‐line managers (*n* = 8) were performed. Furthermore, the user survey (*n* = 33) was administered by staff. Beyond this point, checklists and progress reports were collected for each ward (*n* = 22) during monthly site visits by NSPH (May–December). Fieldnotes further contributed information on context, implementation process and mechanisms of impact. Themes used for interview protocols, survey, monthly reports and fieldnotes were: (1) overall reflection on the implementation in relation to context, (2) routines and collaboration around the organization of the delivery, barriers and facilitators with regard to (3) introduction and follow‐ups (delivery), (4) RG content and usage and (5) need for implementation planning and support. Themes helped to ensure that similar information was collected across data sets. In addition, education was evaluated by means of two global survey questions for attendees: ‘Was education content and RG intervention relevant for your practice?’ and ‘Do you feel ready to use the RG in your practice?’ Focus group interviews with staff lasted 45–60 min,^35^ while individual interviews with managers took 30–40 min. Recorded interviews were transcribed verbatim and the multiline free text answers were transferred into Microsoft Word documents. Fieldnotes and reports (coproduced checklists, progress sheets on delivered and received RG interventions) were documented in Excel Spreadsheets.

Initial data preparation and analysis were performed by authors C. A., J. A. and L. N. (NSPH). The next passage concerned a deductive and summative analysis of secondary data according to the process evaluation components by the first author (U. B.). One spreadsheet for each inpatient ward was created for within‐group analysis, involving triangulation of data. Two rows corresponded to qualitative or quantitative data, respectively, while 10 columns represented data of education, follow‐up at 3 months and eight monthly visits. All spreadsheets were then merged into one comprehensive spreadsheet to allow for in‐between ward comparisons on similarities and differences. Next data, collapsed spreadsheet and interview material, was deemed as belonging to either of the process evaluation components of context, implementation process, or mechanisms of impact. As a final step, analysis and triangulation of data sets were transformed into a coherent text. When no particular stakeholder group is mentioned in the results, all participants are represented. Data treatment steps and analysis were critically reviewed by all authors to mitigate interpreter bias and increase validity.^34^

## 3 RESULTS

### 3.1 Context

During the preparatory phases of the implementation programme (Table 1), all stakeholders perceived the RG intervention as having legitimacy, to be ‘right and acceptable’. One manager mentioned during the individual interviews the following,

No matter what, it is always difficult to introduce something new because of the high workload. But the Recovery Guide is a simple tool, so it feels natural. We like that it is a coherent, it becomes more structured and may facilitate our work. (Manager)

However, during the initial delivery, after the Education Steps, all wards faced implementation barriers. Lack of resources at an organizational (meso) level greatly affected managers' and staff's outlook and possibilities of implementing the RG (microlevel). Wards that were subjected to reorganization had an extreme and unstable workplace with little capacity left for implementing new interventions. Staff felt unwell, lacked energy, and were too busy getting settled (Table 2, suborganization D). The preconception of a high and complex workload among staff was another barrier evident among all wards at first. This assumption was also present during ‘hectic’ months. Emergency situations with users (anger, risk of harm issues and severe side effects) competed with resources needed for delivery. One manager said, ‘There is sense of frustration and stress about not keeping up with the good work of the Recovery Guide’. High staff turnover further limited the capacity to keep resources in terms of educated staff, and temporary staff generally lacked the interest to deliver the RG. High patient turnover also limited opportunities for delivery. A lacking tradition of implementing novel interventions was another initial barrier present at both meso‐ and microlevels of the organization. Monthly documentation conveyed that staff generally lacked interest at first. Some were cautious and felt that the RG was too comprehensive and overwhelming, for them and for the users. Furthermore, beliefs and attitudes about medical treatment and planning did not always fit the recovery values, which constituted another barrier. Staff perceived users as being ‘too ill’ to benefit, in three out of the five psychosis wards, in one addiction ward and in one general ward. Some managers believed that the intervention might be too difficult to implement, with ‘their inpatients’. Reflections like, ‘it was difficult to *make* patients interested in working with the RG’, ‘how will we *make* the patients believe that this is valuable’, ‘how can we *make* patients realise that it is an opportunity’ occurred. This outlook on users' capability affected both managers' and staff's priority, willingness and energy to introduce the RG. In six wards, the initial contextual barriers lasted. One manager explained, ‘staff may be keen on changes and are not reactionaries, yet some interventions are not followed through’ (Manager).

On the contrary, wards that overcame initial barriers had not only managers who were engaged but also RG responsible staff and peer supporters who helped to facilitate solutions, collaboration and shared responsibilities during implementation (microlevel). The manager's perspectives of the RG intervention facilitated quality implementation. One manager said, ‘I am in a position where I can get all the pieces in the puzzle together’. Another facilitator was when managers recognized staff's need for attitude and behaviour change and focused on solutions that could be incorporated into regular routines, meetings and administration. They facilitated discussions about recovery and supported staff through everyday nudging. RG responsible staff functioned as ‘champions’, and some wards shared ‘superchampions’ to facilitate collaborative change. Coproduction at meso‐ and microorganization levels was thus critical for sharing resources and solutions, and for creating overall culture, incentives that facilitated wards' capacity to implement the RG intervention. Peer supporters (rooted in shared understanding and mutual empowerment) further facilitated attitudinal change towards recovery perspectives. Notably, once staff started to deliver the RG and thus gained experience of working ‘with’ the users, their beliefs about mental illness and recovery, and whether implementation was achievable, clearly changed. One staff said in a focus group interview, ‘I believe that everything that benefits the service user is positive’. Staff became essentially positive and perceived the RG as fun to work with, and considered it to be a long‐awaited material and tool to connect with users. This new way of working was perceived as ‘right’ for both staff and users and enhanced their relationship and dialogue since it helped staff to recognize and realize users' point of view and experiences.

The continuous dialogue among NSPH, staff and managers further facilitated implementation. Monthly site visits were decisive for the initiation, resumption and sustainability of the delivery, ‘to keep it alive’. Since coproduction did not cease during the study period, the implementation of the RG could be resumed when contextual barriers could be resolved. For example, five wards set new dates for education (13B, 16B, 20D, 21D, 22D), and another six wards planned continuous education sessions to boost implementation (4B, 8B, 9B, 10B, 15C, 18C), all in the forthcoming year of 2020 (Table 2). Thus continuous support as well as ongoing primary and continued education were in favour of sustainability.

### 3.2 Implementation process

The preceding four phases of the Implementation programme (Table 1) were possible to complete. It demonstrated a buy‐in and commitment from the head of MHS, and that coproduction among stakeholders was possible to implement. During the Reflection phase, staff rated the education as relevant for practice (*n* = 139: completely, 74.8%; partly 24.5%, not at all 0.7%), and felt ready to use the RG intervention (*n* = 139: completely, 57.6%; partly 41.7%, not at all 0.7%). However, with regard to the implementation phases henceforth, the extent to which the 22 wards had the capacity to implement the RG varied. As addressed in Table 2, one ward became a daycare during the period (6B). Subunit D failed to implement due to reorganization. Wards from subunit B (9B, 13B, 16C) made attempts but were too challenged by initial contextual barriers. Yet, 14 wards (64%) delivered the RG intervention throughout the period, although two of them (4B and 8B) did not register data delivery and hand documentation (Table 2).

#### 3.2.1 Dose delivered and received

The dose delivered by staff, that is, how much, when, what of the RG content that was delivered during information and follow‐ups, was neither consistent among wards. As shown in Table 2, the mean rate of delivery in relation to admitted users was 53%, the respective minimum and maximum monthly rates were 0 and 98, and the mean range was 35%. Five wards, irrespective of specialty, managed to introduce the RG at a rate of >50%. The proportion of staff who delivered the material was not reported. Among staff who continued implementation (12 wards), the delivery was perceived as natural, stimulating and meaningful. ‘When’ to deliver the RG intervention, the timing, was not straightforward. In adherence to the manual, delivery was often performed in a flexible and dynamic way, sensitive to users' needs. Staff perceived delivery as a balancing act in relation to regular work tasks. It involved decision‐making and planning about available time and energy in relation to regular work tasks, for example, quiet in the ward, late afternoons, evenings and weekends. Some involved users early on during their stay, in adherence to manual guidelines, in open dialogue about care planning and recovery. Yet, an adaptation was that the delivery occurred at the end of users' stay, in relation to discharge when staff anticipated users to feel better and more settled. At this point, the dialogue between staff and users focused more on discharge issues and less on recovery‐focused treatment and care planning. *What* to deliver varied among staff. Some favoured chapters on users' rights, while others portioned out brief 15 min sessions, while at the same time nurturing an open dialogue about recovery according to user needs. Notably, in wards where implementation was not completed, managers and staff perceived it as difficult to assess when, what part and how much of the content would benefit a service user. At times, the RG material was simply delivered on the bed table (adaptation). Users needed to take initiatives and approach staff themselves.

The quality of the RG material enhanced legitimacy and facilitated delivery. It was considered easy to introduce; ‘it sells itself and is sensible’ (*manager*), ‘it is simple to deliver and does not require much extra work’ (*staff*). All data reflected that the format was appropriate, for example, texture, layout with calm colours and clear division of chapters. The content was considered elaborate and profound and addressed recovery issues in relation to inpatient care and discharge. The voice was kind and nonoffensive, and empty spaces allowed users to interact, comment and reflect on plans and goals. Staff, however, thought that the perspective of being a parent was missing. One staff member mentioned,

It is important to address children's situation in the guide. That there is support to get, so this does not get perceived as scary. (Staff)

The capacity to deliver the RG was facilitated by shared responsibility and coproduction of managers and staff as well as integration of routines and administration. Over time, it was evident that staff became closer and regularly reminded each other about delivery and shared caseload. As an adaptation to individual delivery, many wards started regular group sessions and/or published RG citations on tablets, used on canteen tables. By contrast, in wards where implementation was not pursued (Table 2), it was considered the staff's individual responsibility to deliver. No capacity was built in the organization, and staff more easily lost their focus, interest and forgot about it.

The dose received among users varied and depended on the degree of flexibility and personalization of support. Most users stated that the content was easy to learn and satisfying to use, and worked through all chapters. Some had difficulty absorbing it during the first period of their stay or when they felt unwell. Yet others preferred the RG at the beginning, especially those who were inpatients for the first time. Services users who replied (*n* = 32) rated the *overall impression* to be very good (*n* = 8, 25%), or good (*n* = 21, 66%), while three did not (less good: *n* = 0; bad: *n* = 3, 9%). However, the relevance in relation to *discharge* was not entirely clear (*n* = 32: completely, *n* = 9, 28%; certain extent, *n* = 14, 44%; no, *n* = 7, 22%; do not know, *n* = 2, 6%). The format and pedagogic structure were perceived as thorough, complete and well‐performed, and the majority thought it was completely *comprehensive* (*n* = 13, 39%), or to a certain extent (*n* = 15, 46%), while four said that it was not (*n* = 4, 12%), and one did not know (*n* = 1, 3%). Otherwise, the *clarity* of the RG content was very good (*n* = 7, 21%) or good (*n* = 22, 67%), while two rated less good (*n* = 2, 6%), and two, bad even (*n* = 2, 6%). At first, the material could be perceived as too extensive, and that a lot of efforts was required by the users. However, there was an agreement that ‘once you got into it’ it became useful. The majority (*n* = 32) thought the length was good (*n* = 22, 69%), while some meant it was too long (*n* = 8, 25%), or short (*n* = 2, 6%). According to staff, a certain level of endurance was required and some users lost attention and concentration.

The preferred usage ranged from users being independent and deciding themselves, with brief follow‐ups, to having it delivered to them in a highly structured way with hands‐on support and encouragement from the staff. One user explained in the free‐line answer, ‘After the introduction I mainly used it on my own, to remind me of what is important in life’. Another user wrote, ‘It is required that staff are engaged. If they are, it is good’. Not all chose to write, some only read and reflected. However, this too impacted users' perspectives on themselves and goals ahead. Group sessions with peers were appreciated and lived experience narratives facilitated discussions (often about loneliness) and assimilation. Family, friends and community support were thought as critical for usage.

#### 3.2.2 Reach

Reach, that is, whether the intended target group is reached and comes in contact with the intervention, is reflected in Table 2. There was no systematic difference in reach among wards. The majority of users (*n* = 33) perceived that the RG fitted inpatients completely (*n* = 14, 42%) or to a certain extent (*n* = 15, 46%), while one ticked off ‘I did not know’ (3%) and three users ‘no’ (9%). One user wrote that it may be difficult to reach those with lower cognitive functions and/or reading difficulties. Staff affected reach through their beliefs, that first‐time inpatients benefitted the most. Those who had been previously exposed, been there long, had self‐admitted themselves, or had a psychosis, withdrawal symptoms, or had long‐term treatment plans, for example, residential treatment, supported housing accommodations, were at risk of not being reached. Of note, staff from wards with no regular implementation shared these assumptions. Other staff suggested adaptations to increase reach, a thinner and simpler version, visible tabs to more freely browse, locate and explore and other languages.

### 3.3 Mechanisms of impact

Participants' responses and reflections on the impact of the intervention were categorized as: *A new perspective on mental health, well‐being and recovery; Capacity building of a recovery approach in inpatient settings*; and *A meaningful outlet for users' thoughts and feelings on recovery, sharing narratives and posing goals*.

Delivering the RG intervention provided staff with *A new perspective on mental health, well‐being and recovery*. It changed staffs' outlook and increased positive attitudes around recovery in inpatient care. It provided tangible support and functioned as an ‘ice‐breaker’, a tool for approaching and sharing perspectives, knowledge and decisions with users helped to build staff–user relationships. The enhanced focus on solutions, strengths and well‐being was also mentioned.

*Capacity building of a recovery approach in inpatient settings* concerned developing a neutral language, focusing on mental health, enabling both organized and spontaneous discussions about recovery. Recovery goals were reported in regular treatment planning records. According to managers, building capacity required no extra efforts or costs once initial contextual barriers were resolved, that is, applying new perspectives, finding time and resources, and integrating new regular routines had passed. Instead, it offered quality improvement, for example, new ways of working together and approaching and sharing perspectives and decisions with users.

The RG intervention was reflected by users and staff as *A meaningful outlet for users' thoughts and feelings on recovery, sharing narratives and posing goals*. The quality of the RG facilitated acceptability and was considered a nonoffensive material with space for own narratives, thoughts and feelings, which made it possible to identify needs and make sense of experiences in the past and present. It facilitated a reflection process to safely express expectations and fears, as well as hope for better times. The discharge chapter formed a link to the home, outpatient and community context. The following citations represent five different users.

How great this is! Can you read where you want and write how much you want? (User)

It was great to be able to write it off your system […] I thought about it even before, how important it can be to have an outlet for your thoughts and feelings by writing. Previously, I wrote a diary every day […] some may not dare to talk about their innermost thoughts, so now you can write instead. Then staff can read, if you are up to it. (User)

It helped me to have something meaningful to work with, while waiting for my mental health to get better. (User)

It was very nice that you could have dreams. I think you easily forget that otherwise. I love to travel, and was reminded a lot about that. (User)

This RG should have existed many years ago. The first time I ended up in a ward, in the ‘90s’, I wish it existed then. (User)

Some users became more self‐directed and shared their goals, which at times were integrated into the care plan (adherence to guidelines). According to staff, narratives of others' lived experiences were much appreciated by users, to connect with others.

#### 3.3.1 Mediators

During preparatory phases, critical mediators for change were the (1) perception of acceptability and value of RG intervention, and (2) the buy‐in and coproduction among all stakeholders. During active implementation (start of delivery), several mediators helped to overcome the implementation barriers, that is, (3) engaged managers and staff who believe that benefits outweigh the initial extra workload, (4) ward capacity to integrate delivery into routines and develop a reflective culture of recovery, (5) managers who prompt and nudge staff for desired delivery, and perhaps the most critical mediator, (6) staff who develops experiential knowledge through ‘doing’ the RG intervention with users. Once views and attitudes about recovery changed, (7) staff's interest and willingness to implement the RG became equally important mediators. A fieldnote from a site visit revealed,

From the beginning it was very difficult to support them to get started with their work. Status reports were rarely submitted, and we were told things like ‘our patients do not need to work on recovery’. After the follow‐up in October, staff had completely turned around, and had started groups and become very engaged. Now they are working on just fine. (Fieldnote)

Moreover, the (8) NPSH user organization functioned as a mediator for change during the entire implementation process (Table 1). They enabled coproduction among stakeholders, had a mandate to orchestrate the initial and ongoing support during implementation.

## 4 DISCUSSION

The process evaluation showed that it was feasible to implement the RG intervention in inpatient ward settings, regardless of specialty. When initial contextual barriers were overcome the RG intervention had the potential to provide staff with new perspectives on mental health and recovery, organizational capacity to integrate a recovery approach and users with a meaningful outlet of lived experience to influence treatment goals. The actual delivery provided staff with experiences and tools to ‘do things with users’, to start a conversation, listen, learn, share and plan for care and recovery goals and, to reach common grounds, which previous research has emphasized.^2^, ^12^, ^15^

The components of implementation components were feasible to implement (Table 1) without further adaptations. The initial phases involved structured decision‐making processes among stakeholders that built general implementation capacity, which is in keeping with the planning steps for quality implementation.^30^ At these phases, the perceived legitimacy of the RG intervention was a critical mediator. In theory, there was an overall agreement that the intervention ‘was right’ and service users benefitted from having greater influence. However, our findings revealed how active implementation (delivery) was not possible for all, and out of the 22 wards, 68% succeeded in this performance act (Table 2). This discrepancy between the initial phases and active delivery may be due to it being easier to unite around goals, content and reporting systems, where stakeholders feel involved and enriched, while delivery entails that attitudes, behaviours and routines need to change, using resources not always available or yet in place.^30^ In light of our results, it would thus be wise to take stock over available resources and critical implementation barriers in the local context, as well as to address critical mediators for change and customize implementation strategies at different levels of the MHS organization.

Adaptations are increasingly recognized as an inevitable part of the implementation process and uptake of an intervention.^36^, ^37^, ^38^ In the present study, adaptations were not uncommon. They were related to the contextual barriers present, which is often the case.^39^, ^40^ For example, staffs' stereotypical views and negative outlook on users' capability and recovery made them deliver RG intervention towards the end of users' stay and focus on discharge issues, rather than incorporating recovery values and goals in treatment within the first five days, in adherence with the manual. Likewise, staff tended to direct ‘what’ to deliver, instead of providing an overview and opening up a mutual dialogue about recovery, to empower user influence. However, some adaptations were made to better fit the busy inpatient context without losing the integrity of the intervention, like having group sessions, printing narratives to be used in shared spaces and introducing the RG when it was quiet in the wards. Users appeared to value the benefits of groups, of sharing lived experience narratives with peers. In line with literature in the area,^38^ we conclude that it is crucial to monitor adaptations and adherence in relation to outcome while raising awareness of implementation planning and strategies during implementation efforts to come. Moreover, we stress the importance of having an implementation team function to address these important issues, as previously emphasized.^32^, ^37^ Of note, the NSPH displayed this function, through joint troubleshooting with stakeholders, monitoring and having an open dialogue about implementation and reporting improvements. Such negotiations of objectives we believe fostered collaboration and optimized uptake at both meso‐ and microlevels of the organization.

Sustainability is regarded when adherence to the original intervention seems fair, and the capacity to continue delivery has been developed.^7^, ^41^ While formal fidelity evaluations help to address adherence and internal validity cross‐sectionally, on‐going implementation strategies helped to resolve barriers and facilitate implementation and external validity, which proves critical for sustainability.^7^, ^42^ Within this regard, our study has contributed to the knowledge of how coproduction helps to initiate, maintain and reinforce the use of the RG. Wards could resume implementation at any time. The social process of coproduction seemed to have fostered relationships and trust among stakeholders, which according to Jensen et al.^31^ may have strengthened the interactional uncertainty at both the horizontal and vertical level of the organization and thus sustainability. Regardless of continuous support strategies to mitigate conflicting goals, horizontal uncertainty was stronger in wards that did complete implementation (Table 2). Common to these wards was that each staff member had to bear his or her own responsibility. Staff may have hesitated, been uncertain and/or reluctant to change, especially since recovery‐oriented services may challenge norms and values of traditional MHS care context.^1^ Divergent views, attitudes, beliefs and behaviour among staff are well‐known implementation barriers.^30^, ^32^ Nonetheless, similar initial challenges in other wards revealed that after a period of horizontal uncertainty the experiential knowledge of social reciprocity with users ‘made the penny drop’. Carrying out the RG intervention facilitated change and a new understanding that fostered engaged staff. Most importantly, managers needed to become mediators for change, to help turnover staff intentions and decision‐making by everyday nudging for desired perceptions and behaviour,^43^ to integrate the intervention into ward routines, and other coproduction efforts. It has long been recognized that the perception of managers mediates workplace outcomes,^44^ and plays a key role in horizontal uncertainty.^6^

The MRC framework provided helpful guidance for planning, analyzing and reporting our study, which strengthens transferability and comparability across process evaluations.^23^ The triangulation between qualitative and quantitative data sets from all stakeholders is a notable strength,^33^ showing that it was possible to implement the RG regardless of inpatient context. However, no purposive sampling procedure of users was performed, which we consider a limitation. Anonymized user data should thus be interpreted with caution and be viewed as a means to triangulate vital data on the lived experience of participation during the inpatient stay. More rigour and emphasis on user perspectives and impact of usage are therefore critical to research. Lastly, we want to emphasize the importance of our choice to use a coproduction approach, which we believe enhanced quality development of the RG intervention and uptake of interventions that matter for users when sharing power and decision‐making during the inpatient stay.

## 5 CONCLUSION

The process evaluation provided an understanding and shed light on the complexity of the translation, the interplay of the intervention and its delivery across 22 inpatient ward contexts. The guide and what has been learned from its ambitious widespread dissemination can contribute to the formation of new perspectives on mental health, capacity in inpatient wards to develop a recovery approach and opportunities for users to share narratives and reflect on recovery and meaningful goals. Mediators helped to improve the effective uptake and fit of the RG interventions to inpatient wards. Coproduction created horizontal and vertical certainty, which implicated sustainability. This knowledge may inform future implementation not only of the RG in similar contexts but also the design and performance of a randomized trial, that is, draw valid hypotheses and include outcomes that reflect mechanisms of impact.^23^

## CONFLICTS OF INTEREST

The authors declare no conflicts of interest.

## AUTHOR CONTRIBUTIONS

All authors have significantly contributed to the present study. Ulrika Bejerholm and David Roe conceptualized and designed the research study. Conny Allaskog, Jessica Andersson and Linda Nordstöm completed the implementation programme and contributed to data collection and initial analysis. Ulrika Bejerholm produced the first draft. All authors supported each other in their respective processes and contributed to the final version of the manuscript.

## Data Availability

The data that support the findings of this study are available from the corresponding author upon reasonable request.
